# Role of Prophylactic Steroids in Differentiation Syndrome

**DOI:** 10.7759/cureus.29531

**Published:** 2022-09-24

**Authors:** Jakia Sultana, Jui Dutta, Sadia Mustarin, Proma Dey, Aditi Roy, Md Y Mamoon

**Affiliations:** 1 Medicine, Comilla Medical College, Cumilla, BGD; 2 Medicine, Mymensingh Medical College, Mymensingh, BGD; 3 Internal Medicine, Chittagong Medical College, Chattogram, BGD; 4 Medicine, Sher-e-Bangla Medical College, Barisal, BGD; 5 Internal Medicine, Queens Hospital Center, New York, USA

**Keywords:** arsenic trioxide (ato), all-trans retinoic acid (atra), prophylactic steroid, acute promyelocytic leukemia (apml), differentiation syndrome

## Abstract

Acute promyelocytic leukemia (APML) is defined as a balanced chromosomal translocation between chromosomes 15 and 17 t(15;17)(q24;q21), which results in the formation of promyelocytic leukemia-retinoic acid receptor-alpha (PML-RARA) fusion protein. A widespread recommendation for APML treatment is combined all-trans retinoic acid (ATRA)/arsenic trioxide (ATO) therapy. Differentiation syndrome (DS), or retinoic acid syndrome, is one of the well-known complications of APML treated with ATRA or ATO. The presenting symptoms of APML-induced DS are diverse, and rare symptoms are easily misdiagnosed. However, unexplained fever, dyspnea, weight gain > 5 kg, leukocytosis, acute renal failure, and a chest radiograph demonstrating pleural or pericardial effusion are the most common manifestations of DS. Early recognition and prompt initiation of corticosteroids are key factors in the management of DS. As soon as ATRA/ATO therapy is started, prophylactic treatment with steroids has been recommended to minimize the severity of DS. It is proposed that ATRA/ATO should be stopped or held once the signs and symptoms of DS develop. This case report describes a 45-year-old male who was diagnosed with APML after he developed episodes of hematuria and nose bleeding at home. The patient was also given an empiric steroid along with ATRA/ATO to lessen the intensity of DS. Our study suggests that early initiation of prophylactic steroid treatment can improve the prognosis and mortality of patients with APML-induced DS.

## Introduction

Acute promyelocytic leukemia (APML), a type of acute myeloid leukemia (AML), is characterized by a balanced reciprocal translocation between chromosomes 15 and 17. APML causes a fusion transcript to be produced that connects promyelocytic leukemia (PML) and retinoic acid receptor-alpha (RARA) genes [1,2]. At the promyelocytic stage, this fusion protein blocks the differentiation of leukemic cells [3]. In 1980, all-trans retinoic acid (ATRA) was first introduced for the treatment of APML. ATRA/arsenic trioxide (ATO) treatment shows an improvement in remission rate of 90% and a cure rate of 80% [4]. Prompt initiation of ATRA and ATO can cure APML. This treatment causes apoptosis and differentiation of leukemic cells [1].

Differentiation syndrome (DS)/retinoic acid syndrome, a life-threatening complication of ATRA or ATO therapy, occurs due to systemic inflammatory response syndrome (SIRS) [5]. Approximately 2-48% of APML patients receiving ATRA develop DS, but the rate could be low [6]. DS is characterized by dyspnea, fever, weight gain of >5 kg, peripheral edema, hypotension, acute renal failure, interstitial pulmonary infiltrates, and a leukocyte count greater than 10 x 10^9/L [7]. Severe DS is diagnosed when four or more of the above symptoms are present [8]. Early recognition of DS along with corticosteroid treatment can lead to decreased morbidity and mortality in these patients [6,7]. Some studies also suggest stopping ATRA and ATO if DS develops [7].

In this article, we report an interesting case of a patient with APML who developed moderate DS with prompt induction of steroids that reduced the severity of DS.

## Case presentation

A 45-year-old African American male with no past medical history presented with new-onset, nontraumatic hematuria that started on the day of presentation. He reported extensive bleeding requiring 15 minutes of pressure after a small cut under his nose while shaving at a barbershop two days ago. He had no previous history of bleeding or bruising, and his blood coagulation profile was normal last year.

Clinical examination exhibited normal findings except for swelling of the right eye and blood-crusted nares. His vital signs were stable during admission. After admission, a complete blood count (CBC) hemogram, a metabolic panel, cell morphology labs, and urine routine examination were ordered. He was found to have pancytopenia, and the blood coagulation profile was unusual. Abnormalities were notable for WBC count at 5.14 x 10^9/L (18% monocytes, 12% promyelocytes, 15% myelocytes, and an absolute neutrophil count of 0.29). Furthermore, the hemoglobin level was 7.5 g/dL, the platelet count was 27,000, the serum lactate dehydrogenase level was 329 IU/L, the prothrombin time was 16.8 seconds, the fibrinogen level was 91, and the D-dimer level was 26,900 ng/ml. Moreover, fluorescence in situ hybridization (FISH) and peripheral blood and bone marrow samples were sent to the laboratory for morphologic, immunophenotypic, cytogenetic, and molecular analysis to confirm the diagnosis. Peripheral blood film (PBF) results manifested atypical lymphocytes with possible blasts with Auer rods. Furthermore, the FISH result was consistent with t(15,17) PML/RARA. Therefore, the patient was diagnosed with a case of APML with disseminated intravascular coagulation (DIC).

The patient was started with treatment of ATRA 40 mg twice daily and IV ATO 10.6 mg daily. At the same time, he was also treated with allopurinol, cefepime, and vancomycin. Liberal blood product support with fresh-frozen plasma, cryoprecipitate, and platelet transfusions was immediately started.

To prevent the development or decrease the severity of DS, prednisone 0.5 mg/kg was started as prophylaxis from day one of starting ATRA through induction. Close monitoring was continued to observe any other complications like DS, tumor lysis syndrome, pseudotumor cerebri, headache, and hepatotoxicity from ATRA treatment. Concurrently, the patient was also monitored carefully to determine whether he developed fever, weight gain, hypotension, dyspnea, any radiographic opacities, pleural or pericardial effusion, or acute renal failure, signifying the development of DS.

The next day after starting the ATRA therapy, the patient developed a fever (38.5°C/101.3°F), mild shortness of breath, and swelling in the bilateral legs. He had gained ~6 kg since admission. Hence, the lab investigations were done again. It was found that after starting ATRA/ATO therapy, the WBC count increased from 9.8 x 10^9/L to 28 x 10^9/L, and on the third day, the WBC reached a maximum of 40 x 10^9/L. Oxygen saturation was observed at 95% on room air. However, the CXR, as well as ECG, were normal.

Considering the risk of DS, ATRA and ATO were both discontinued. Hydroxyurea was administered promptly for cytoreduction. Along with broad-spectrum antibiotics, intravenous dexamethasone 10 mg 12 hourly was started on the third day. After seven days of steroid therapy, when the patient's signs and symptoms began to subside, the dose of dexamethasone was tapered to 10 mg in the AM and 5 mg in the PM. Tapering was completed within 14 days.

The patient's absolute neutrophil count (ANC), platelet count, and hemoglobin slowly increased after the dexamethasone therapy. The patient tolerated ATRA/ATO well at this point with minimal symptoms. The patient's ANC was persistent in the 2000s, and he was stable for discharge. Continued outpatient care on ATRA/ATO with a plan for hemato-oncology follow-up after three days was advised to discuss consolidation therapy further.

## Discussion

DS mainly occurs after the introduction of ATRA and ATO. This may be due to a systemic inflammatory response by proinflammatory cytokines interleukin (IL)-1, IL-6, IL-8, and tumor necrosis factor released after the introduction of ATRA. There is also increased vascular permeability and endothelial damage due to cathepsin G [6,9]. All these lead to sign symptoms related to DS. After the initiation of ATRA, DS usually occurs within two to 21 days [9]. Late presentations may also happen [8].

The clinical presentation of DS is widely variable, ranging from moderate to severe. Patients may present with fever, dyspnea, hypotension, weight gain > 5 kg, peripheral edema, acute renal failure, and pulmonary infiltrates or pleural or pericardial effusion may be observed on chest imaging [6,7]. Weight gain > 5 kg is considered a red flag of developing DS, so it should be monitored [6]. If three or more sign symptoms are present, DS can be confirmed; however, one or more features are sufficient to begin empiric treatment of DS [6].

Guidelines for empiric treatment of DS include dexamethasone 10 mg every 12 hours; if no improvement is seen, the frequency should be increased and tapered once the sign symptoms are completely resolved [6]. Different guidelines give different opinions about ATRA continuation. In one article, it is suggested that ATRA and ATO should be retained if no improvement occurs after initiation of dexamethasone or if severe DS develops, and it can be restarted once the symptoms resolve [6]. Another suggests stopping ATRA immediately if DS is suspected [7]. There is no specific guideline to start prophylaxis for it, but prophylaxis with prednisone or dexamethasone should be considered if WBC > 5 x 10^9/L [6].

A study by Wiley and Firkin showed a reduction of pulmonary complications from DS in patients with WBC > 10 x 10^9/L [10]. The PETHEMA LPA99 trial, where systemic prednisone was used for prophylaxis in all patients irrespective of WBC count, showed a reduced incidence of severe DS in comparison to the LPA96 trial, where prophylaxis with dexamethasone was given to patients with WBC > 5 x 10^9 only (11.3% vs. 16.6%; P = 0.07) [10]. Prophylaxis can be given with prednisone [11-14], methylprednisolone [15], and dexamethasone [16,17] during the entire duration of induction therapy [11,13] or for a limited period of time, ranging from five to 15 days [12,14,15,17] in patients treated with ATRA plus ATO or ATRA plus chemotherapy.

Steroids decrease alveolar epithelial cell chemokine production, so there is less flow of APML cells in the alveolar epithelial cells, as found in vitro and in vivo studies [10]. These may prevent the development of severe DS as there is reduced chemokine production in alveolar epithelial cells and prevention of pulmonary infiltration of APML cells after steroid use. As alveolar chemokine secretion takes place in an earlier stage of development of DS and steroids do not affect a later stage of DS development where chemokine production occurs from differentiating APML cells, we should start steroids in the early phase of DS, preferably as prophylaxis [18].

Our patient, a 45-year-old African American male, came with new-onset nontraumatic hematuria and extensive bleeding from the nose. After admission, we found pancytopenia with WBC of 5.14 x 10^9/L, and PBF showed atypical lymphocytes with a possible blast with Auer rods that suggest APML. Later, we confirmed the diagnosis with FISH, which was consistent with t(15,17) PML/RARA. We started ATRA at 40 mg twice daily and IV ATO at 10.6 mg daily with prophylaxis prednisone 0.5 mg/kg [5]. On the second day of starting ATRA, the patient developed fever, mild shortness of breath, peripheral edema, weight gain > 6 kg, and a WBC count of 9.8-28 x 10^9/L. DS was suspected, and ATRA was discontinued [7]. A chest X-ray was done as the patient developed shortness of breath. It was found to be normal.

Our findings suggest that prednisone in patients with DS prevented the progression to severe respiratory distress, evidenced by normal chest X-ray and oxygen saturation, and minimized the severe symptoms of DS.

## Conclusions

In APML, ATRA and ATO treatment regimens lead to excellent remission and survival rates. DS is a frequent and life-threatening complication in patients treated with ATRA and ATO. In our case report, we gave prophylaxis of steroids to the patient along with ATRA and ATO induction therapy that decreased the severity of DS. When ATRA and ATO regimen is initiated for treatment of APML, a prophylactic steroid should be used as standard therapy.
